# Lessons from fatal re-expansion pulmonary oedema: case series

**DOI:** 10.1093/icvts/ivab366

**Published:** 2021-12-28

**Authors:** Dambuza Nyamande, Siphosenkosi Mazibuko

**Affiliations:** Sefako Makgatho Health Sciences University, Pretoria, South Africa

**Keywords:** Re-expansion pulmonary oedema, Pleural effusion

## Abstract

The goal of this study was to investigate the extent of the alveolar-capillary membrane porosity in patients with severe re-expansion pulmonary oedema. The biochemistry of airway fluid of two patients who died of re-expansion oedema was compared to their blood biochemistry. The airway fluid was comparable to plasma, while no blood cells were observed across the alveolar-capillary membrane. The membrane was linked to a fishnet that traps cells on one side, while plasma sieved through.

## INTRODUCTION

Re-expansion pulmonary oedema is a rare form of acute lung injury, occurring as a complication of sudden lung re-expansion after pleural drainage [1]. This potentially fatal condition can occur when more than 1.5 L of fluid is drained rapidly [2]. However, the pathophysiology of this condition remains unknown. Our case series is a direct comparison between airway fluid and blood, to demonstrate the extent of the alveolar-capillary membrane permeability in patients with extensive re-expansion pulmonary oedema.

## CASE PRESENTATIONS

### Case 1

**Figure 1:**
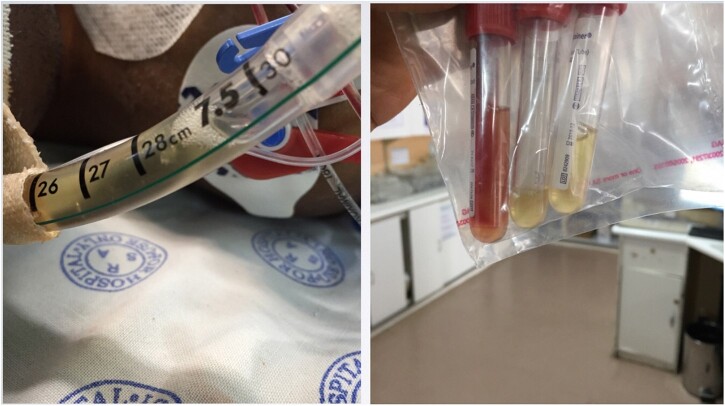
Airway fluid that accumulated in the endotracheal tube of patient 1 (left image). The colour of airway fluid was comparable to plasma seen at the bottom of the first tube (image on the right).

A 44-year-old patient presented with a loculated right empyema thoracis from transdiaphragmatic rupture of a right subphrenic abscess. After failed thoracentesis coupled with a demonstrable visceral pleural cortex on chest computed tomography scan, a single-stage pleural drainage and lung decortication were done via thoracotomy. About 3000 ml of thick pus was drained from both the chest and abscess, and the lung re-expanded fully after decortication. Large amounts of frothy serosanguineous fluid were noted in the patient’s endotracheal tube. Bronchoscopy revealed large amounts of the fluid pouring from left and right bronchial trees. Despite being on a ventilator, 400 ml of airway fluid was suctioned in about 15 min and even more thereafter. Ineffective ventilation due to build-up of fluid in the endotracheal tube resulted in hypoxic cardiac arrest and death (Fig. 1). The airway fluid and patient’s blood were sampled and their properties were compared (Table 1).

**Table 1:** Biochemical comparison between plasma and airway fluid of patients in both cases

	Case 1		Case 2	
Parameter (units)	Airway fluid levels	Plasma levels	Airway fluid levels	Plasma levels
Sodium (mmol/l)	137.0	136.0	146.0	154.0
Chloride(mmol/l)	97.0	100.0	112.0	102.0
Urea (mmol/l)	4.7	4.7	4.0	4.4
Creatinine (µmol/l)	73.0	70.0	66.0	70.0
Total protein (g/l)	31.0	31.0		
Albumin (g/l)	8.0	7.0		
Cholesterol (mmol/l)	0.4	0.5		
Albumin (g/l)			21.0	28.0
Lactate dehydrogenase (mmol/l			413.0	446.0

### Case 2

A 31-year-old male patient presented with a massive right pleural effusion. A chest drain was inserted, draining 800 ml of straw-coloured fluid. The drain was clamped with the aim of draining smaller amounts in a controlled fashion. Unfortunately, the clamp slipped off resulting in sudden drainage of 1500 ml, causing pulmonary oedema that required ventilation. The endotracheal tube rapidly filled up with large amounts of serosanguineous fluid, again resulting in death. The airway fluid and blood biochemical properties were compared (Table 1).

## DISCUSSION

First described in 1853 by Pinault [3], this complication can occur after drainage of a pneumothorax. Literature review on re-expansion pulmonary oedema case reports reveals a 42% mortality rate [4]. Reperfusion injury theory, and subsequent inflammatory response, may explain the development of bilateral pulmonary oedema in some cases [5].

Direct comparison between airway fluid and blood biochemical components in our cases demonstrated a massive protein leak through the capillary alveolar barrier. Total protein, albumin and lactate dehydrogenase levels were comparable to serum (Table 1). Both small (electrolytes) and large molecules (proteins) in airway fluid were comparable to plasma levels, while no blood cells were observed in the fluid. Therefore, the capillary alveolar membrane was leaky to the plasma components but not to cellular blood components. In our view, the membrane’s behaviour was analogous to a fishnet, trapping the fish (blood cells) on one side while non-cellular blood components sieved through (fishnet theory).

Hydrostatic mechanism was previously demonstrated in 5 out of 7 patients by Sue *et al.* [6]. Using the same airway to plasma protein ratio of >0.65, our study demonstrates an increased capillary permeability mechanism. Unlike our study, the airway fluid sampled using a catheter wedged in the distal bronchus demonstrated blood cells.

As shown in our study, ineffective ventilation due to fluid accumulation in the airway is fatal and extracorporeal membrane oxygenation should be considered.

## CONCLUSION

Our study demonstrated an alveolar-capillary membrane that is permeable to plasma, but not to blood cells, in some patients with severe re-expansion pulmonary oedema. Although larger studies have been previously reported, this study adds some understanding to the potentially complex pathophysiological mechanisms of this condition.

## ETHICAL STATEMENT

Written informed consent was obtained from both patients relatives for publication.

**Conflict of interest:** none declared. 

## DATA AVAILABILITY STATEMENT

The data underlying this report will be shared upon request to the corresponding author.

## REVIEWER INFORMATION

Interactive CardioVascular and Thoracic Surgery thanks Paola Ciriaco, Francoise Le Pimpec-Barthes and the other anonymous reviewers for their contribution to the peer review process of this article.
